# Utilizing small-angle X-ray microdiffraction to explore neurodegeneration in human brain tissue

**DOI:** 10.1063/4.0000976

**Published:** 2025-10-27

**Authors:** Prakash Nepal, Abdullah Al Bashit, Lee Makowski

**Affiliations:** Northeastern University, Boston, MA, USA

## Abstract

The power law behavior (*I α q-p*) seen in the small angle regime of X-ray microdiffraction data from human brain tissue offers a unique opportunity to reveal insights into molecular pathology of disease process in Alzheimer’s disease (AD). The scattering exponent, p, obtained from the slope of *log(I)-log(q)* plot over a range that extends from 0.007 to 0.1 Å-1 along with uncorrelated SAXS and WAXS intensities revealed, surprisingly, the existence of sub-micrometer sized voids in fixed, dehydrated thin tissue sections. The heterogeneous distribution of voids across the tissue may act as a biomarker to distinguish the regions with significant neurodegeneration.

Using scanning X-ray microdiffraction, we mapped the pathological hallmarks i.e. voids and lesions across brain regions affected by neurodegenerative disease at different stages. The abundance and size of voids were correlated to the regions associated with neurodegeneration and severity of disease. These observations were complemented by silver-stained images and conventional histology providing a novel multi-faceted window for characterizing these neurodegenerative processes. Overall, these interpretations of data across the brain regions involving in the trajectory of disease contribute a better understanding of disease progression.

